# Arctic Insects Show a Highly Dynamic Microbiome Shaped by Abiotic and Biotic Variables

**DOI:** 10.1007/s00248-025-02685-z

**Published:** 2026-01-10

**Authors:** Sara Nørris Christoffersen, Stine Karstenskov Østergaard, Nadieh de Jonge, Cino Pertoldi, Jesper Givskov Sørensen, Natasja Krogh Noer, Torsten Nygård Kristensen, Jeppe Lund Nielsen, Simon Bahrndorff

**Affiliations:** 1 Department of Chemistry and Bioscience, Aalborg University, Fredrik Bajers Vej 7H, 9220 Aalborg, Aalborg East Denmark; 2 Aalborg Zoo, Aalborg, Denmark; 3 Department of Biology, Aarhus University, Aarhus, Denmark; 4 ENORM Biofactory, Flemming, Denmark

**Keywords:** Arctic insects, Diet, Endosymbiont, Microbiome, Humidity, Symbiosis, Temperature

## Abstract

Arctic regions are inhabited by terrestrial ectotherms that have adapted to an extreme environment where food resources are limited. The host associated microbiome may partly explain their ability to live under these conditions, but very little is known about the microbiome of Arctic ectotherms. We investigate how the bacterial community of the Greenlandic seed bug (*Nysius groenlandicus*) and damsel bug (*Nabis flavomarginatus*) is affected by different abiotic and biotic factors (time, acclimation temperature, humidity, and diet) under both field and laboratory conditions. We found large differences in the bacterial composition and diversity between the two species including species-specific presence of potentially symbiotic bacteria. The bacterial community of both species changed across the season, which may be explained by the changing climatic conditions, such as temperature and humidity. This was further supported by results from the laboratory experiments. We also found that diet changed the bacterial composition in both species and that bacteria could be transferred from prey to predator. Together, these results show that the bacterial community of some Arctic insects are highly dynamic and modulated by different abiotic and biotic factors, suggesting that the microbiome plays an important role for these organisms to persist in an extreme and resource-limited Arctic environment.

**Supplementary Information:**

The online version contains supplementary material available at 10.1007/s00248-025-02685-z.

## Introduction

Terrestrial ectotherms in the Arctic environment are faced with some of the harshest conditions on our planet, with long cold winters and short summers with periodically high temperatures [1, 2]. Arctic regions are also very dry with low availability of water during winter because it is typically frozen [3]. Additionally, water limitation is predicted to become an increasing problem for terrestrial ectotherms in polar regions during summer droughts [4–6]. These extreme environmental conditions can strongly influence the fitness of individual organisms [7] and are important drivers of evolutionary changes in Arctic species [8]. Not only are climatic factors limiting terrestrial ectotherm life cycles, but the limited availability of some food resources further complicates insect development during the brief summer season in the Arctic [7]. These constraints on the development of terrestrial ectotherms imposed by limited food resources become an even greater challenge during summertime when metabolism, and therefore nutritional demand, is higher due to high microhabitat temperatures [8, 9]. The energy expenditure is even more challenging for univoltine arthropods that must optimize growth, development and reproduction during the short Arctic summers [1, 8].

Insects and their associated microbiome can make up a close relationship [10], such as in termites [11], though this is not the case for all insects (see e.g. [12]). Nonetheless, it is well documented that the microbiome can affect responses to pathogens and predators [13] as well as nutrient availability [10, 14] in some insect hosts. In the latter case, the microbiome has been shown to be essential for nutrient provisioning, digestion of carbohydrates, proteins, and lipids, as well as detoxification of xenobiotics [15]. As such, the microbiome can influence which food resources the host can utilise and thus shape the ecological niche of the host [10]. Many insects have adapted to ecological niches with nutrient-poor or refractory food sources in which the nutritional symbiosis between the gut microbiome and the insect host likely played an important role [16]. Because of this, the gut microbiome also differs between species that are adapted to different diets and can even vary between individuals of the same species if they are fed different diets [16, 17]. For herbivorous insects, the gut microbiome contains specialised bacteria, archaea, viruses, and eukaryotes that are involved in the breakdown of recalcitrant plant material [16], and for sap-sucking insects, the gut microbiome contains specific microorganisms that supply the host with amino acids not present in the sap [18]. For some predatory insects, the gut microbiome is involved in metabolizing chemical defence molecules from their prey [19] and microbes may even be acquired from the prey [20]. Furthermore, richness of the gut microbiome seems to be linked to prey diversity as shown in the lady beetle (*Coleomegilla maculata*) and can vary with landscape and environmental variables [20, 21]. Other biotic factors that can affect insect microbiomes, other than diet, include the host species and genotype, feeding behaviour, different symbiotic relationships, vertical and horizontal transmission, and species interactions (e.g. host-host interactions) [22–25].

While the interplay between the microbiome and its role in host metabolism has been widely studied, less is known about the influence of the microbiome on the host’s response to abiotic factors [26]. Studies suggest that the microbiome can have an impact on the host’s thermal tolerance [27, 28] and desiccation resistance [29], and in doing so, the gut microbiome can for example affect the thermal range in which their hosts can survive [27]. There are several mechanisms through which the gut microbiome may aid the host when exposed to stressful temperatures [26], including protection through secondary symbionts [30], modulating host gene expression [31], and affecting host behaviour [32]. For example, *Rickettsia* can induce an expression of genes in its host that are involved in stress responses, thus inadvertently providing the host organism with a higher heat tolerance once exposed to higher temperatures [27, 31]. The gut microbiome can also impact its host in coping with changing habitats by affecting cuticle thickness and melanisation, both factors contributing to desiccation resistance [29]. However, it is possible that exposure to different abiotic factors simply shifts community composition, leading to an overabundance of few bacterial groups in the microbiome [33].

While it has been shown that the gut microbiome of arthropods can play an essential role in how some arthropod hosts respond to both biotic and abiotic factors (see e.g. Corbin et al. [27], Engl et al. [29] & Renoz et al. [34]), the number of studies on how the microbiome affects Arctic and Antarctic terrestrial arthropods are few [35]. This is surprising given that both abiotic factors (e.g. fluctuating temperatures and humidities) and biotic factors (e.g. limited resource availability) historically have shaped Arctic terrestrial arthropod communities [1]. For example, recent results have shown how thermal tolerance is affected by both temperature and humidity of the surroundings even on short time scales [36], thus affecting survival of the organisms. It is currently unclear how the bacterial community composition changes on a temporal scale in Arctic arthropods and how it is shaped by different abiotic and biotic factors. Therefore, data from these regions is much needed and may provide important information about host-microbiome interactions and how it affects survivability in the extreme Arctic environment.

The aim of this study is to investigate how the bacterial community of an herbivore insect, the Greenlandic seed bug (*Nysius groenlandicus*), and a predator, the damsel bug (*Nabis flavomarginatus*), is affected by different abiotic and biotic factors (time, temperature, humidity, and diet). To do this, we collected individuals directly from the field to investigate both daily and seasonal differences in bacterial community composition within and between species and how this relates to changes in temperature and humidity, as well as conducted laboratory experiments to address the influence of acclimation temperature, humidity, and diet on the bacterial community under controlled conditions. We hypothesise that (1) the bacterial community of the two species will reflect the feeding strategy of the species (e.g. carnivory or herbivory). As such, we expect that *N. groenlandicus*, being a herbivore living on a nitrogen poor diet, will possess microbes that provide nutrients as has been found in other herbivorous species [16], and that *N. flavomarginatus*, being a generalist predator living on diverse prey items, will exhibit a diverse bacterial community due to ingestion of prey microbiomes [20, 21]. We also hypothesise that (2) the bacterial community composition of *N. groenlandicus* will change throughout the sampling season [37]. Furthermore, we hypothesise that the bacterial community will change when the two species are exposed to different acclimation (3) temperatures and (4) humidities as these factors have been shown capable of changing the microbial composition [27, 29]. Lastly, we further hypothesise that (5) the bacterial community will change depending on the diet that *N. groenlandicus* and *N. flavomarginatus* are fed [17]. Additionally, we expect the presence of groups of bacteria with association to endosymbiosis that may be adaptive for their insect hosts living in an extreme Arctic environment [16, 34].

## Materials and Methods

### Study Site and Organisms

The Greenlandic seed bug *Nysius groenlandicus* inhabits Arctic and sub-Arctic regions and is one of the most dominant insect species in grassland, where it can reach numbers exceeding 100 individuals per square meter [38]. *Nabis flavomarginatus* is a generalist predator observed to prey on *N. groenlandicus* and is found in grassy vegetation in sub-Arctic regions [38]. Both species occur naturally in large numbers in the area where the experiment was conducted. Here, the thermal conditions of their habitats can vary widely, with local microhabitat temperatures reaching over 30 °C during the day and subzero temperatures at night during summer [8]. Both species are univoltine and complete their life cycle within the short Arctic summer season [38].

Individuals of *N. groenlandicus* and *N. flavomarginatus* were collected from the area in and around Narsarsuaq, South Greenland (61°09′30.5"N 45°25′20.9"W) in July–August of 2021 (experiment 1 and 3) and 2023 (experiment 2, 4, and 5). Individuals were collected by net sweeping in the field and transported back to the laboratory in vials; *N. groenlandicus* were transported in plastic vials (9.5 × 2.5 cm) covered with a foam plug with 15–20 individuals per vial and *N. flavomarginatus* were transported in individual 4 mL screw-cap glass vials (4.5 × 1.5 cm). Permits allowing collection of animals were obtained in accordance with Home Rule Greenland regulations (licence no. G23-006 (Nanoq—ID nr.: 22142249) and G21-012 (Nanoq—ID nr.: 18401054)). This study did not require animal ethics approval.

The food sources used for the diet scenarios in experiment 5 were collected in the field on the same day as the experiment was commenced. Individuals of *N. flavomarginatus* were fed with *N. groenlandicus* and the leafhopper *Psammotettix lividellus*, which were collected by net sweeping in the field and transported back to the laboratory in separate plastic vials (9.5 × 2.5 cm) covered with foam plugs and contained 15–20 individuals per vial. Individuals of *N. groenlandicus* were fed with seeds from *Angelica archangelica* and *Juncus trifidus*, which were likewise collected in the field and transported back to the laboratory in small plastic containers where they were kept refrigerated until use.

#### Microhabitat Temperatures and Humidities

Temperature loggers (TMS-4, TOMST, Czech Republic) were placed at the sites where the animals were collected around Narsarsuaq, and air, soil, and ground level temperatures as well as soil moisture content were continuously recorded throughout the sampling period.

### Study Design

To investigate the differences in bacterial community composition between *N. groenlandicus* and *N. flavomarginatus*, as well as how different abiotic and biotic factors (time, temperature, humidity, and diet) affect the bacterial composition, five different experiments were conducted. In experiments 1 and 2, individuals were collected directly from the field to determine the effect of both daily (*N. groenlandicus* and *N. flavomarginatus*) and seasonal (*N. groenlandicus*) environmental variations on the bacterial community. In experiments 3, 4, and 5, the effects of acclimation temperature, humidity, and diet, respectively, were investigated in controlled laboratory experiments.

#### Species Differences in Bacterial Community Composition Across Days (experiment 1)

In the first experiment, individuals of *N. groenlandicus* and *N. flavomarginatus* were collected in the field on 20/08–2021 (hereafter named “day 1”) and 24/08–2021 (hereafter named “day 5”). These collection timepoints represented a day with cloud cover and small temperature fluctuations (day 1) or a day with sun and no cloud cover with large temperature fluctuations (day 5) [39]. Individuals were collected at the same time of day on the two sampling days. This was done to see if there were differences between species and to assess what effect climate (temperature and humidity) in the field prior to sampling had on the gut microbiome. Individuals used for the experiment were sexed in the field and transported back to the laboratory within 30–45 min of collection where they were transferred to 70% ethanol. Five replicates of ten individuals for *N. groenlandicus* and five replicates of three individuals for *N. flavomarginatus* were collected on both day 1 and day 5, respectively. Only females of both species were used in the experiment.

#### Seasonal Variation in Bacterial Community Composition (experiment 2)

To further elucidate what effect seasonal variation and change in field temperature and humidity had on the bacterial community in *N. groenlandicus,* individuals were collected from the field throughout the summer season (24/07–2023 to 20/08–2023) approximately every fourth day, resulting in samplings from eight non-consecutive days in total. Individuals were collected on each sampling day, both in the morning (around 10 a.m.) and evening (around 6 p.m.). Field collected individuals were transported back to the laboratory within 30–45 min of collection where they were sexed and subsequently transferred to 70% ethanol. Five replicates of five individuals were collected at each sampling time on each sampling day. Only females were used in the experiment.

#### The Effect of Acclimation Temperature on Bacterial Community Composition (experiment 3)

Following experiment 1 and 2, we wanted to assess what effect different constant acclimation temperatures had on the bacterial community in *N. groenlandicus* and *N. flavomarginatus*. Eight replicates of five individuals of *N. groenlandicus* were acclimated at three different temperatures (10.2, 15.0, and 19.6 °C) and four replicates of three individuals of *N. flavomarginatus* were acclimated at six different temperatures (2.5, 4.5, 10.2, 15.0, 19.6, and 25.2 °C). For both species, acclimation was 24 h at the respective acclimation temperatures. Field collected individuals were transported back to the laboratory within 30–45 min of collection where they were sexed and subsequently transferred to the respective acclimation temperatures. Only females of both species were used in the experiment. Acclimation was done in a water bath where animals were submerged in the water in glass vials (4.5 × 1.5 cm). *N. groenlandicus* were exposed in groups of five individuals per vial whereas *N. flavomarginatus* were placed individually in each vial due to a larger body size. No water or food was supplied during acclimation. The animals were transferred to 70% ethanol following the 24 h of acclimation where *N. groenlandicus* were pooled in four replicates of ten individuals and *N. flavomarginatus* were pooled in four replicates of three individuals.

#### The Effect of Humidity on Bacterial Community Composition (experiment 4)

In experiment 4 we wanted to assess the effect of humidity on the bacterial community in *N. flavomarginatus* in a controlled laboratory experiment. Five replicates of three individuals were exposed to five different relative humidities (5, 90, 96, 98, and 100% RH) at 15.6 °C for 24 h. Field collected individuals were transported back to the laboratory within 1–2 h of collection where they were sexed. Only female individuals were used in the experiment. To obtain a relative humidity of 100% throughout the experiment, 50 mL of tap water was added to 365 mL lid covered plastic containers. To obtain a relative humidity of 90, 96, and 98% RH, NaCl solutions of 166.3 g/L, 71.2 g/L, and 35.8 g/L, respectively, were produced and 50 mL of a given solution was added to 365 mL plastic containers covered with a lid as described elsewhere [40]. For 5% RH 40 g of dehydrated silica gel (corresponding to the same volume as 50 mL of water) was added to 365 mL lid covered plastic containers. During exposure, individuals of *N. flavomarginatus* were held in individual mesh-covered glass vials, but in bundles of three vials per plastic container. After 24 h of exposure to a given humidity, individuals were pooled into five replicates of three individuals and transferred to 70% ethanol. No food or water was supplied during the experiment.

#### The Effect of Diet on Bacterial Community Composition (experiment 5)

In experiment 5 we wanted to assess if diet had an impact on the bacterial community in *N. groenlandicus* and *N. flavomarginatus*. Three different diet scenarios were investigated for both species. Five replicates of seven *N. groenlandicus* were subject to either feeding with seeds of *A. archangelica*, *J. trifidus*, or no feeding at all. For *N. flavomarginatus,* fifteen individuals were subject to either feeding with *N. groenlandicus*, *P. lividellus*, or no feeding at all. Field collected individuals were transported back to the laboratory within 1–2 h of collection where they were sexed and only female individuals were used for both species. For *N. groenlandicus,* 105 individuals were distributed in groups of 7 individuals per Petri dish (14 × 2 cm) and for *N. flavomarginatus,* 45 individuals were each placed in their own Petri dish. Water was supplied to all Petri dishes by filling a 1.5 mL microtube with water and closing with a ball of cotton wool. A round sheet of paper was supplied for each Petri dish containing a *N. flavomarginatus* to create a better surface for predation. The animals were starved for 24 h at 16.0 °C for *N. flavomarginatus* and 15.1 °C for *N. groenlandicus*. After 24 h of starvation, animals were subject to 24 h of feeding on different diet scenarios at the same temperature. Following the feeding, all animals were transferred to 70% ethanol. *N. groenlandicus* were transferred in the original five replicates of seven individuals per treatment, while *N. flavomarginatus* were pooled into five replicates of three individuals per treatment.

### Microbial Analysis

The samples collected in 2021 (experiments 1 and 3) and 2023 (experiments 2, 4, and 5) were prepared, pre-processed, and analysed in largely the same way, but with slight deviations due to advances in sequencing technology during this time period.

#### DNA Extraction and 16S rRNA Gene Amplicon Sequencing

DNA was extracted using DNeasy® Blood & Tissue kit (Qiagen GmbH, Hilden, Germany) and the Purification of Total DNA from Insects protocol in accordance with the manufacturer’s specifications. DNA concentrations were measured with Qubit™ 1 × dsDNA HS Assay Kit (Invitrogen, USA) on Qubit 3 fluorometer (Invitrogen, USA).

The full length 16S rRNA gene of samples collected in 2021 was amplified using the primer 27F: 5’-AGAGTTTGATCCTGGCTCAG-3’ [41] and 1492R: 5’-GGTTACCTTGTTACGACTT-3’ [42]. The PCR-reaction was conducted in duplicates of 25 μL (PCRBIO 1 × Ultra Mix (PCR BIOSYSTEMS), 400 nM of each primer, 10 ng of template DNA, and nuclease-free water). Amplification was run under following conditions: an initial denaturation at 95 °C for 2 min, followed by 35 cycles of 15 s at 95 °C, 15 s at 55 °C and 90 s at 72 °C and a final elongation at 72 °C for 5 min. A positive and negative control were included to ensure the quality of the amplicon generation. The libraries were purified using CleanNGS (CleanNA, The Netherlands) with a sample:bead ratio of 1:0.8 and the library concentration was measured as previously described. The size of selected libraries was checked by Agilent 2200 TapeStation using ScreenTape D1000 (Agilent Technologies, USA).

Two hundred fmol of PCR product from each sample was pooled and subsequently barcoded, pooled equimolarly, DNA repaired and end-prepped, adapter ligated, cleaned and loaded onto a single MinION R9.4.1 using the SQK-LSK110 with the EXP-PBC096 following the manufacturer’s recommendations (Oxford Nanopore Technologies, United Kingdom). The library sequenced for 40 h.

The samples collected in 2023 were prepared like the 2021 samples with minor adjustments according to sequencing technology advancements. Minor adjustments included targeting the V1 (5’-AGRGTTYGATYMTGGCTCAG-3’) [41] to V8 (5’-GACGGGCGGTGWGTRCA −3’) [43] region of the 16S rRNA gene. The size of the samples was checked on an Agilent 4150 TapeStation using ScreenTape D5000 (Agilent Technologies, USA), prepared as described above and loaded onto PromethION R10.4.1 flow cells using the SQK-LSK114 with the EXP-PBC096 in accordance with manufacturer’s recommendations (Oxford Nanopore Technologies, United Kingdom). The library sequenced for 16 h.

#### Bioinformatic Processing

The raw reads from the 2021 dataset were basecalled using Guppy v6.0.6 (https://community.nanoporetech.com) and demultiplexed using Porechop v0.2.3 with the parameters –discard_middle, –require_two_barcodes, and –barcode_threshold 85 [44]. Raw reads from 2023 were basecalled and demultiplexed using Dorado v.0.5.0 with sup v.4.3.0 (https://github.com/nanoporetech/dorado) using standard settings in the MinKNOW software with the addition of required barcodes in both ends. Amplicon pre-processing was conducted using the ONT-AmpSeq pipeline [45] with Q-score = 11 (2021 data) and Q-score = 20 (2023 data) and filtered according to their respective lengths. Adequate sequencing depth was achieved for all experiments (Fig. S1).

Sequences were clustered into Operational Taxonomic Units (OTUs) with 97% sequence similarity. Taxonomy was assigned based on the SILVA database [46] and OTUs missing information on genus level were run through the blastn algorithm, BLAST + v2.15.0 [47] and assigned further taxonomic information when appropriate. OTUs with less than 5 total reads and OTUs representing chloroplasts and mitochondria were removed from the dataset.

### Data Analysis

Statistical analyses were performed in R version 4.4.2 [48] using RStudio version 2024.12.0.467 [49] and the following packages: *ampvis2* version 2.8.9 [50], *car* version 3.1.3 [51], and *vegan* version 2.6.10 [52].

Alpha-diversity of the bacterial community was investigated by looking at the number of unique OTUs present as well as Shannon’s Diversity Index [53]. To assess the effect of the different variables investigated in the experiments on the alpha-diversity, one- or two-way ANOVAs were performed on experiment 1, 2, and 3, while a Kruskal–Wallis was performed on experiment 4 and 5. This was done due to the data being normally distributed in the former experiments, while the data in the latter was not. A significance level (α) of 0.05 was chosen. The effect of the chosen environmental variables on the beta-diversity was investigated with a Constrained Correspondence Analysis (CCA) [54, 55] and with Bray–Curtis PERMANOVAs with subsequent beta-dispersion tests [56]. Bray–Curtis distances were visualised via NMDS. Bacterial community composition was further investigated using heatmaps showing the 15 most abundant OTUs and their relative abundances (see Andersen et al. [50]).

To assess the effect of temperature and humidity in the field prior to sampling on bacterial diversity in experiment 2, a multiple stepwise regression with bidirectional selection [57, 58] was conducted after removing variables with high collinearity based on their Variance Inflation Factor [59]. The initial climate variables included were average, minimum, and maximum measurements of temperature and humidity during either 4 or 8 h prior to sampling. Selection of the best regression model was done based on the Akaike Information Criterion (AIC) [60].

## Results

### Species Differences in Bacterial Community Composition Across Days (experiment 1)

**Fig. 1:**
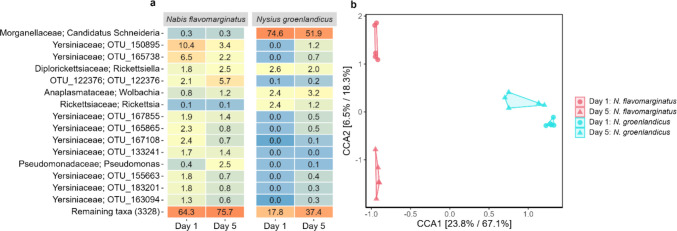
Effect of species and sampling days on bacterial composition. **a** Heatmap of the relative abundance of the top 15 shared OTUs (at family and genus level) between *Nabis flavomarginatus* and *Nysius groenlandicus* in experiment 1, faceted by sampling day. **b** Canonical Correspondence Analysis (CCA) for experiment 1

In total, 3802 unique OTUs were observed for the two species collected across two days in the field. Of those, 1312 OTUs (34.5% of the total number of unique OTUs) were unique for *N. flavomarginatus*, 1190 OTUs (31.3%) were unique for *N. groenlandicus*, and 1300 OTUs (34.2%) were shared between the two species. The most abundant OTUs were also different between the two species (Fig. 1A). Most of the OTUs in the dataset belonged to the order *Enterobacterales* (87.9%) followed by *Rickettsiales* (1.2–4.9%). The two most abundant families in the dataset were *Morganellaceae* and *Yersiniaceae* (Fig. 1A), which both belong to *Enterobacterales*. *Candidatus* Schneideria, belonging to the *Morganellaceae* family, was the most predominant genus found in the microbiota of *N. groenlandicus*, consisting of between 51.9% and 74.6% of the total abundance (Fig. 1A). The trend was less clear for *N. flavomarginatus*, with representatives from the family *Yersiniaceae* being the most predominant group (between 15.4% and 40.7% of the total abundance). Other bacterial groups were also found in the two species including the genera *Wolbachia*, *Rickettsiella*, and *Rickettsia* in *N. groenlandicus*, and *Pseudomonas*, *Rickettsiella*, and *Wolbachia* in *N. flavomarginatus* (Fig. 1A). For both species, a relatively large number of all OTUs, and the reads they represent, could not be assigned to specific taxa at the genus level, ranging from 14.2% to 56.7% unassigned OTUs per sample for *N. groenlandicus* and 89.4% to 98.5% for *N. flavomarginatus*.

In general, the tendency was that the bacterial diversity increased across days for both species. For *N. groenlandicus*, the total number of unique OTUs increased from 665 on Day 1 to 991 on Day 5, and the Shannon diversity index increased from 4.6 on Day 1 to 5.6 on Day 5. For *N. flavomarginatus*, only the Shannon diversity index increased across days from 5.5 on Day 1 to 5.9 on Day 2. This was further supported by a two-way ANOVA showing that sampling day had a significant effect on bacterial diversity both when looking at unique OTUs (*p* < 0.05, *n* = 20, *F* = 4.8) and the Shannon index (*p* < 0.001, *n* = 20, *F* = 20.5), and that species also had an effect on the bacterial diversity when looking at the Shannon index (*p* < 0.01, *n* = 20, *F* = 12.9). A Canonical Correspondence Analysis (CCA) confirmed that the bacterial community composition indeed was different between the two species and differed between the two sampling days (Day 1 and Day 5) for each species (Fig. 1B). It also showed that the distance between sampling days was bigger for *N. flavomarginatus* compared to *N. groenlandicus* (Fig. 1B). However, while the PERMANOVA revealed that there was a significant difference in the bacterial communities between the two species (*F* = 22.4, df = 1, *p* < 0.001), sampling day was not significant (*F* = 2.1, df = 1, *p* = 0.09) (see Fig. S2A).

### Seasonal Variation in Bacterial Community Composition (experiment 2)

**Fig. 2:**
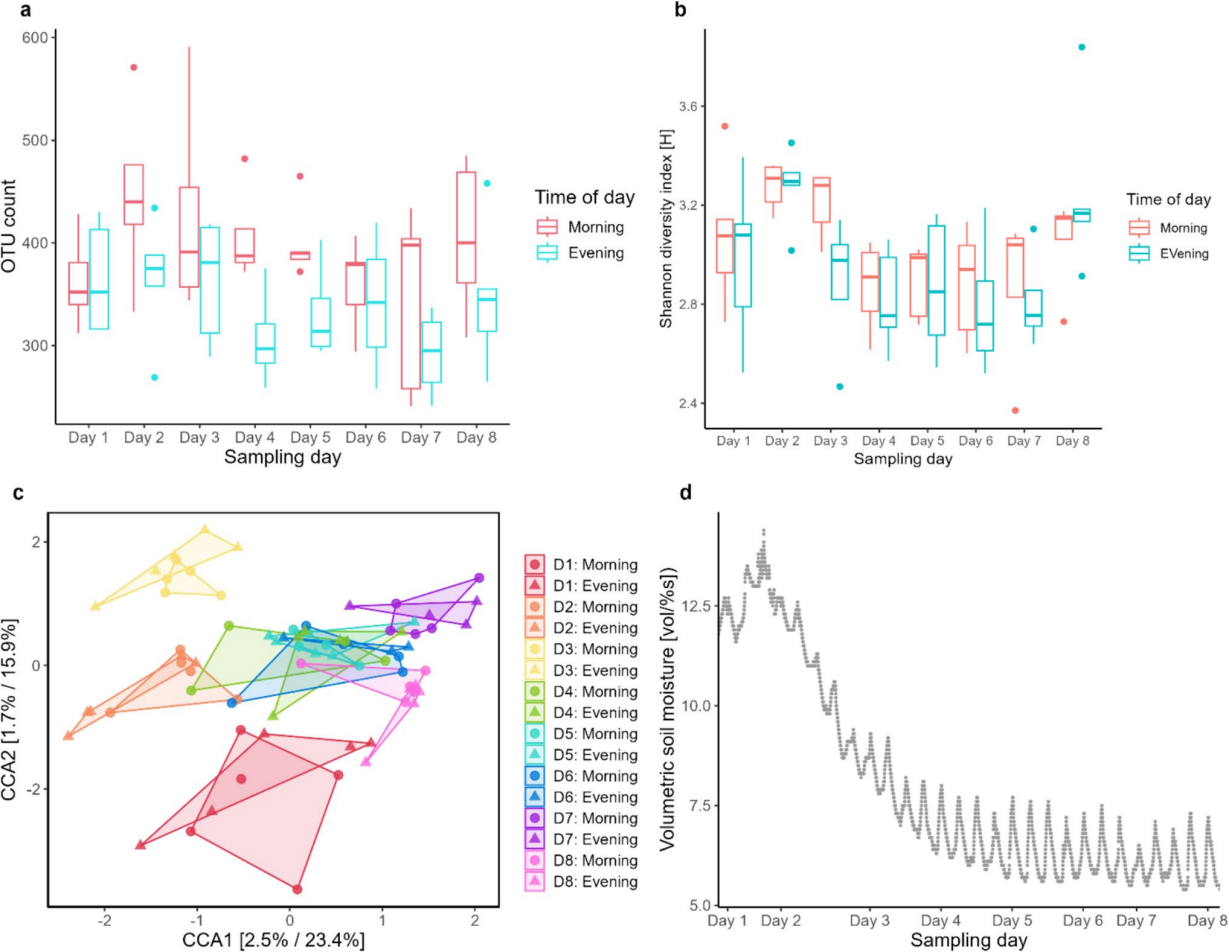
Effect of sampling day and sampling time on bacterial composition. Bacterial diversity as measured by the number of unique OTUs **a** and the Shannon diversity index **b** for *Nysius groenlandicus* across the sampling period in experiment 2, with samplings roughly every four days. **c** Canonical Correspondence Analysis (CCA) for experiment 2. **d** Soil humidity measured in the field during the sampling period of experiment 2

Some additional bacterial groups were detected in *N. groenlandicus* in experiment 2, including *Spiroplasma, Candidatus* Karelsulcia, and *Enterococcus* (Fig. S3). In this experiment, there was a relatively low percentage of OTUs that could not be assigned to taxa at the genus level, ranging from 3.2% to 9.4% across samples. The bacterial diversity of individuals of *N. groenlandicus* generally differed across sampling days and showed some variation within each sampling day between morning and evening samplings when looking at number of unique OTUs (Fig. 2A). When looking at the Shannon diversity index, however, the diversity was generally similar between morning and evening samples (Fig. 2B). A two-way ANOVA showed that sampling day had a significant effect on the bacterial diversity when measured with the Shannon index (*p* < 0.001, *n* = 79, *F* = 4.5), but the time of day did not (*p* > 0.05, *n* = 79, *F* = 1.1). The exact opposite trend could be seen from a two-way ANOVA performed on number of unique OTUs, with time of day having a significant effect on diversity (*p* < 0.001, *n* = 79, *F* = 14.4), while sampling day was not significant (*p* > 0.05, *n* = 79, *F* = 1.6). A CCA showed that some sampling days (Day 1, 2, 3, and 7) clustered independently compared to other days (Fig. 2C). The CCA also showed that morning and evening samplings clustered together for each individual day (Fig. 2C). However, the PERMANOVA revealed that there was a significant difference between groups for both sampling day (*F* = 2.5, df = 7, *p* < 0.001) and time of day (*F* = 3.9, df = 1, *p* < 0.01) (but see Fig. S2B).

The bacterial diversity, as measured by the Shannon diversity index, followed the same tendency as the humidity at the site of collection (Fig. 2D). Both showed an increase from Day 1 to Day 2 followed by a decrease throughout the rest of the sampling period, reaching an all-time low at Day 7 (Fig. 2B, 2D). The same tendency could not be seen for the measured temperatures in the field (Fig. S4). Using a regression analysis on the number of unique OTUs (R^2^ adj. = 0.19, *F* = 9.7, RSE = 63.4 on 74 DF), the lowest recorded temperature (*p* < 0.001, *t* = −4.2) and the average humidity (*p* < 0.01, *t* = 2.9) in the 8 h prior to sampling had a significant effect. Conversely, when looking at the Shannon diversity index (R^2^ adj. = 0.26, *F* = 7.8, RSE = 0.24 on 72 DF) the lowest recorded temperature both during the 4 h (*p* < 0.05, t = 2.3) and 8 h prior (*p* < 0.01, t = −2.7) to sampling had a significant effect, as well as the minimum (*p* < 0.05, *t* = −2.4) and maximum (*p* < 0.001, *t* = 5.0) recorded humidity during 8 h prior to sampling.

### The Effect of Acclimation Temperature on Bacterial Community Composition (experiment 3)

In experiment 3, a total of 4007 unique OTUs were observed across the two species tested. Of those, 988 OTUs (24.6% of the total number of unique OTUs) were unique for *N. flavomarginatus*, 1129 OTUs (28.2%) were unique for *N. groenlandicus*, and 1890 OTUs (47.2%) were shared between the two species. Thus, like in experiment 1, the bacterial community composition was distinct for each species in this experiment, with the appearance of several of the same bacterial groups including *Wolbachia* and *Rickettsia* (Fig. S5). *Spiroplasma* also appeared in very low abundances (< 0.001%). Similar to the results from experiment 1, the percentage of OTUs that were unassigned at the genus level was higher for *N. flavomarginatus* (ranging from 81.4% to 97.5% per sample) than *N. groenlandicus* (ranging from 25.3% to 83.2% per sample).

**Fig. 3:**
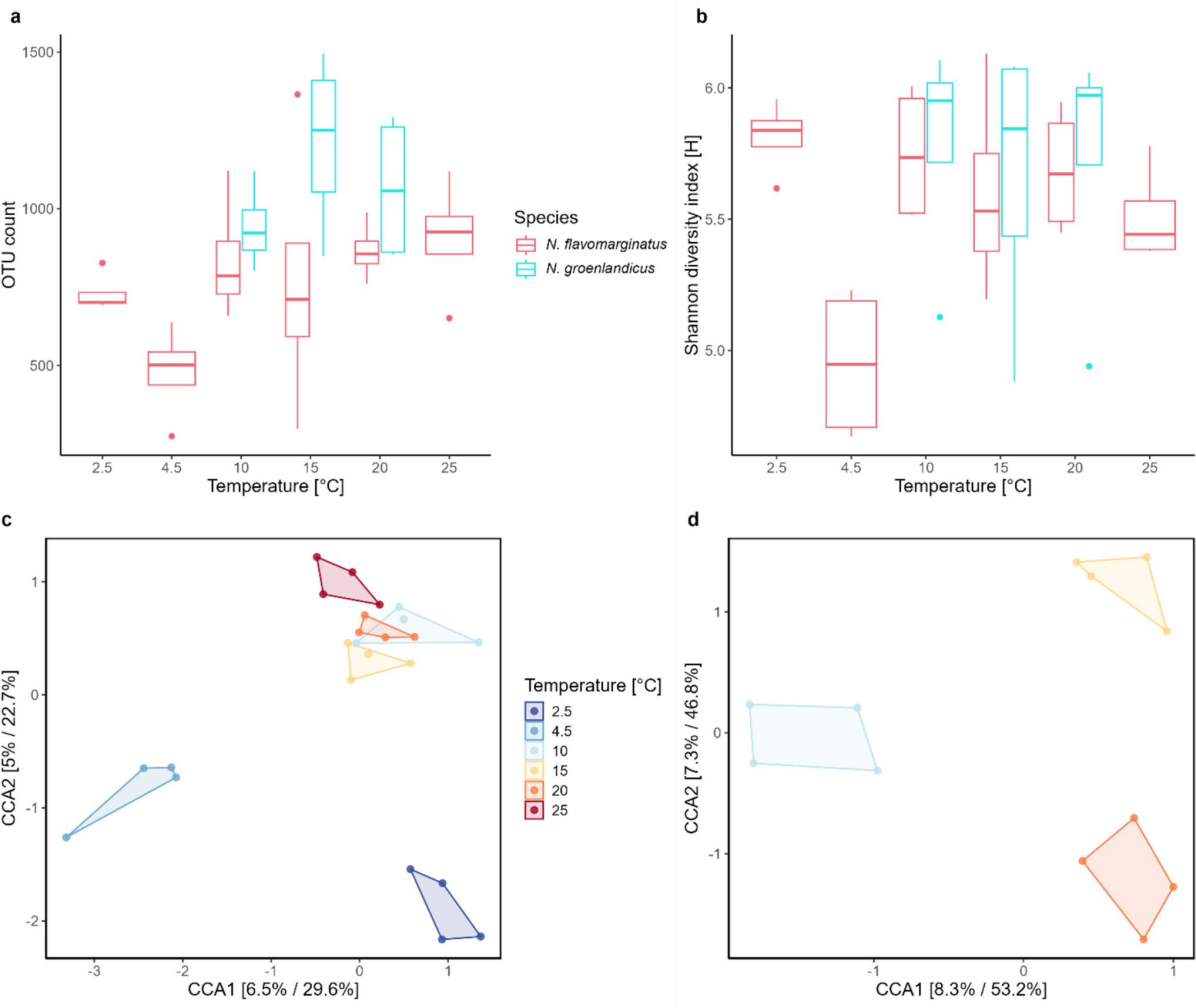
Effect of acclimation temperature on bacterial composition. Bacterial diversity as measured by the number of unique OTUs **a** and the Shannon diversity index **b** for both *Nabis flavomarginatus* and *Nysius groenlandicus* across all temperatures in experiment 3. Canonical Correspondence Analysis (CCA) for *N. flavomarginatus* **c** and *N. groenlandicus* **d** in experiment 3

For *N. flavomarginatus*, the number of unique OTUs increased as the acclimation temperature increased (Fig. 3A), while the only noticeable difference in diversity when looking at the Shannon diversity index was found between 4.5 °C and all other temperatures (Fig. 3B). For *N. groenlandicus,* there did not seem to be a difference in diversity between acclimation temperatures for either number of unique OTUs or the Shannon diversity index, however this species did seem to have a slightly more diverse bacterial community compared to *N. flavomarginatus* (Fig. 3A-B). This was further supported by a two-way ANOVA which showed that both acclimation temperature (*p* < 0.05, *n* = 36, *F* = 3.4) and species (*p* < 0.05, *n* = 36, *F* = 7.1) had a significant effect on the bacterial diversity when measured by unique OTUs, while only temperature (*p* < 0.05, *n* = 36, *F* = 3.3) had a significant effect on the diversity when measured by the Shannon diversity index.

A CCA showed that for *N. flavomarginatus* some acclimation temperatures (10, 15, and 20 °C) grouped more closely together while the more extreme temperatures (2.5, 4.5, and 25 °C) grouped more independently compared to the rest of the temperatures (Fig. 3C). For *N. groenlandicus*, all acclimation temperatures grouped independently (Fig. 3D), but a PERMANOVA showed that this trend was only significant for *N. flavomarginatus* (*F* = 1.3, df = 5, *p* < 0.05) (see Fig. S2C).

### The Effect of Humidity on Bacterial Community Composition (experiment 4)

**Fig. 4:**
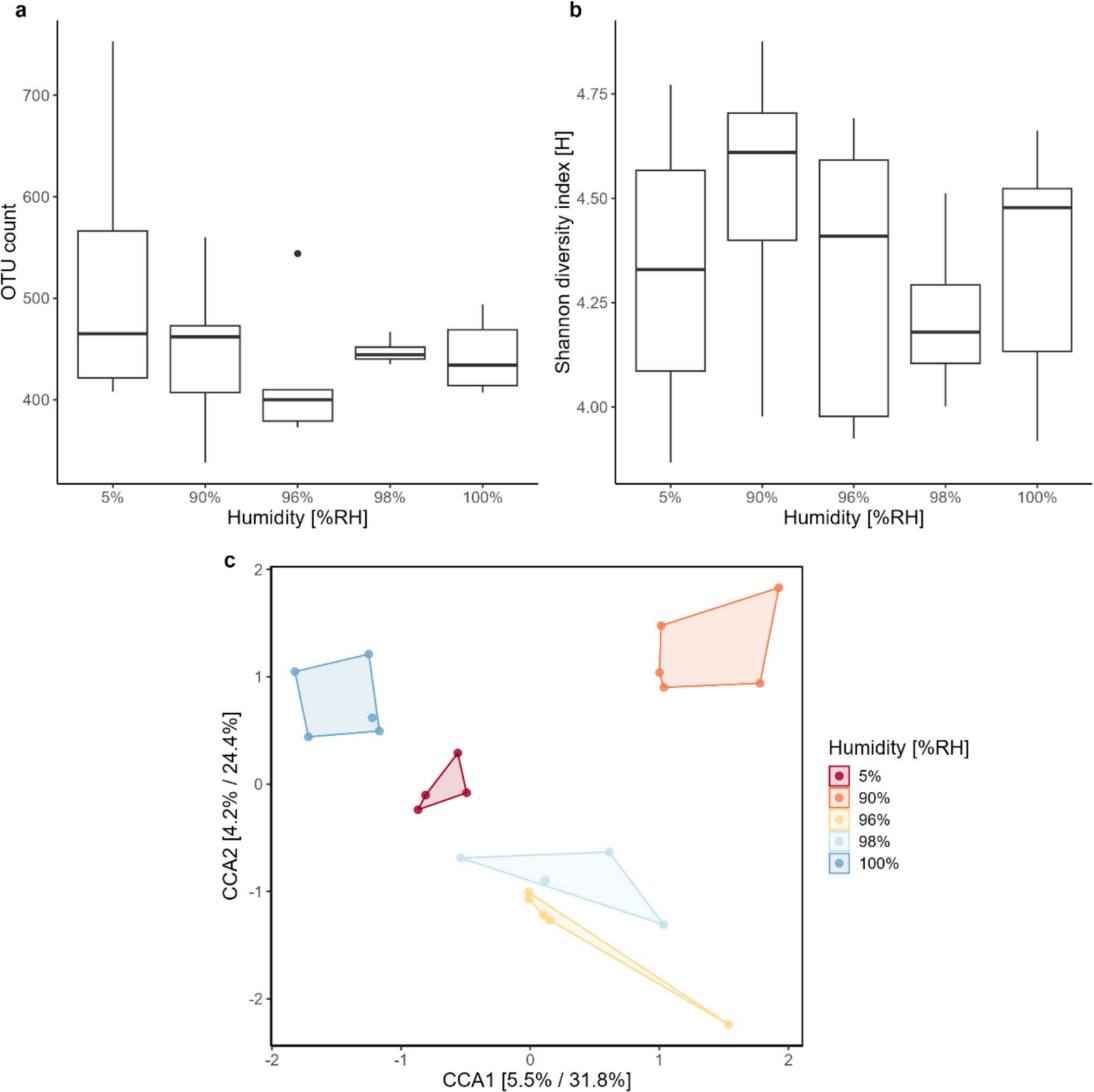
Effect of humidity on bacterial composition. Bacterial diversity as measured by the number of unique OTUs **a** and the Shannon diversity index **b** for *Nabis flavomarginatus* across all humidities in experiment 4. **c** Canonical Correspondence Analysis (CCA) for experiment 4

In this experiment, the percentage of OTUs that were unassigned at the genus level ranged from 38.4% to 66.3%. There was large variation between humidities and no clear trend in the bacterial diversity of *N. flavomarginatus* between these, both when looking at number of unique OTUs (Fig. 4A) and the Shannon diversity index (Fig. 4B). A Kruskal–Wallis likewise showed that humidity had no significant impact on the bacterial diversity for either metric (OTU: *p* > 0.05, df = 4, χ^2^ = 3.2. Shannon: *p* > 0.05, df = 4, χ^2^ = 1.8). However, a CCA, constrained by humidity, showed that 96% and 98% clustered somewhat close together while the rest of the humidities (5, 90, and 100%) clustered more independently compared to the rest (Fig. 4C), suggesting potential differences in bacterial composition between humidities though this was not supported by a PERMANOVA (*F* = 0.8, df = 4, *p* = 0.9) (see Fig. S2D).

### The Effect of Diet on Bacterial Community Composition (experiment 5)

**Fig. 5:**
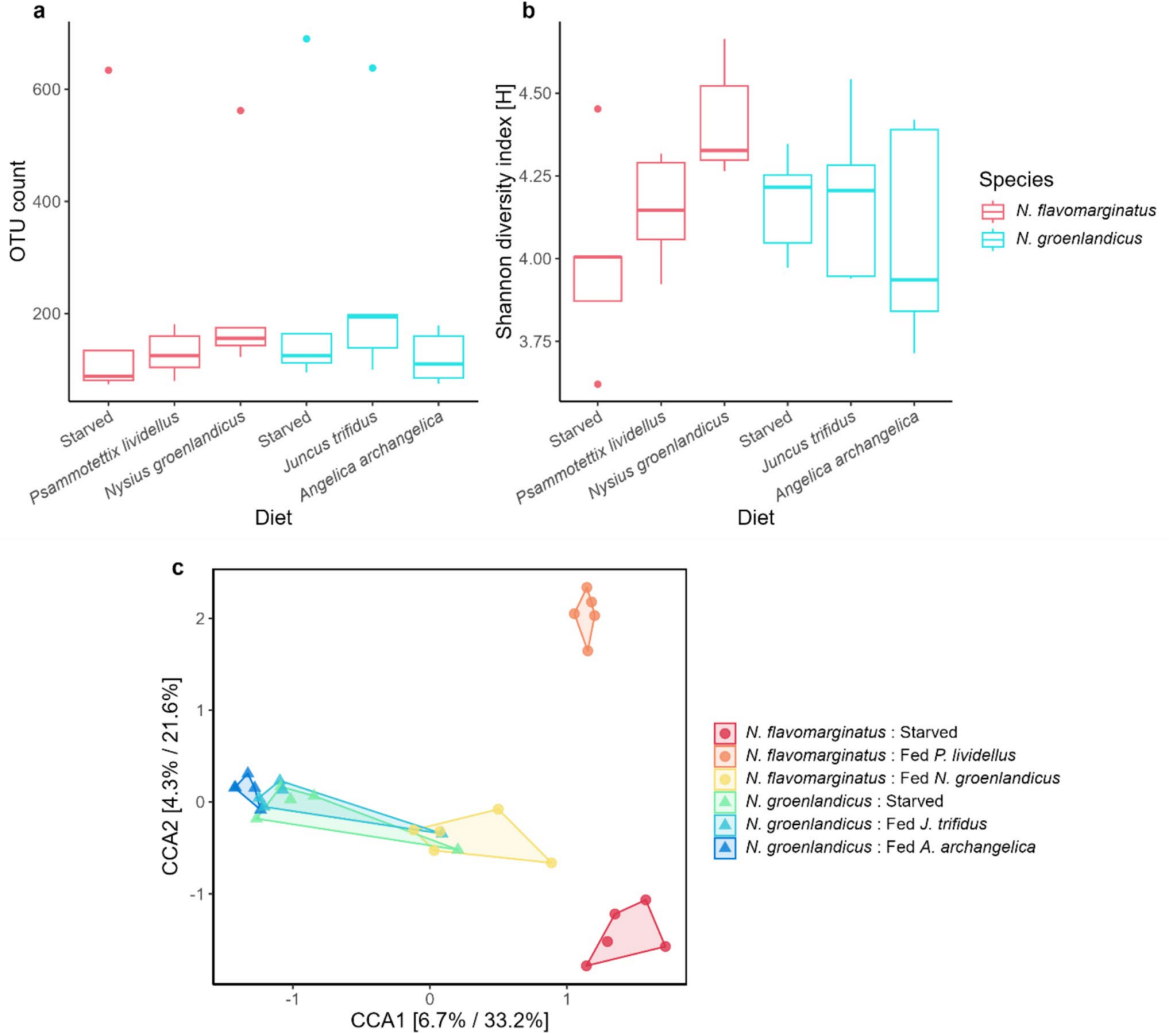
Effect of diet on bacterial composition. Microbial diversity as measured by the number of unique OTUs **a** and the Shannon diversity index **b** for both *Nabis flavomarginatus* and *Nysius groenlandicus* across all diets in experiment 5. **c** Canonical Correspondence Analysis (CCA) for both *N. flavomarginatus* and *N. groenlandicus* in experiment 5

In experiment 5, a total of 1324 unique OTUs were observed across treatment groups. The percentage of OTUs that were unassigned at the genus level was higher for *N. flavomarginatus* (ranging from 26.6% to 64.0% per sample) than *N. groenlandicus* (ranging from 14.0% to 58.3% per sample). Of those, 203 OTUs (15.3% of the total number of unique OTUs) were unique for *N. flavomarginatus*, 201 OTUs (15.2%) were unique for *N. groenlandicus*, and 920 OTUs (69.5%) were shared between the two species (Fig. S7). We found that 12% of OTUs were shared between *N. groenlandicus* and *N. flavomarginatus* that both had been starved, while there was a higher overlap (15%) between starved *N. groenlandicus* and *N. flavomarginatus* that had been fed *N. groenlandicus* indicating that bacteria may have been transferred from the prey to the predator (Fig. S8). For *N. flavomarginatus*, the bacterial diversity varied depending on the diet (Fig. 5A-B). This was supported by a Kruskal–Wallis test showing that diet had a significant effect (*p* < 0.05, df = 2, χ^2^ = 6.0) on bacterial diversity when measured by the Shannon diversity index (Fig. 5B). For *N. groenlandicus*, we did not observe a significant difference in bacterial diversity between diet types (Fig. 5A-B) and a Kruskal–Wallis test likewise showed no significance (OTU: *p* > 0.05, df = 2, χ^2^ = 2.7. Shannon: *p* > 0.05, df = 2, χ^2^ = 0.7).

A CCA, constrained by diet, showed that samples of *N. flavomarginatus* and *N. groenlandicus* clustered separately from each other (Fig. 5C), again highlighting the different bacterial compositions of the two species. Notably, the *N. flavomarginatus* that were fed with a diet of *N. groenlandicus* grouped close to the other *N. groenlandicus* samples compared to the *N. flavomarginatus* that were fed other diets (starved and fed with *P. lividellus*) (Fig. 5C), signifying some overlap in bacterial composition between predator (*N. flavomarginatus*) and prey (*N. groenlandicus*). There was also a larger variation between samples of *N. flavomarginatus* compared to samples of *N. groenlandicus*, suggesting that diet has a larger effect on the microbiota of *N. flavomarginatus* (Fig. 5C). This trend also appeared when looking at a CCA for each species where samples of *N. flavomarginatus* (Fig. S9A) clustered more independently compared to *N. groenlandicus* (Fig. S9B). This was supported by a PERMANOVA where the diet was significant when grouping the two species together (*F* = 1.6, df = 5, *p* = 0.01), but it was not significant when looking at *N. groenlandicus* (*F* = 0.7, df = 2, *p* = 0.8) or *N. flavomarginatus* (*F* = 1.4, df = 2, *p* = 0.09) separately (see Fig. S2E)*.*

## Discussion

Polar terrestrial ectotherms have adapted to the extreme environment of the Arctic and Antarctic, where resources such as food can be limited [7, 8]. Part of the ability of insects to survive here may be explained by their associated microbiome that can affect the ability of the host to cope with different abiotic and biotic variables (e.g. [26]). However, only few studies have investigated the microbiome in Arctic (e.g. ticks [61, 62]) and Antarctic (e.g. midges [63]) terrestrial arthropods under natural conditions and how different biotic and abiotic variables shape the microbiome and in turn affect the ability of these organisms to survive in the polar regions [34].

### Species and Seasonal Variation in Bacterial Community Composition of Field-collected Individuals

We compared the bacterial composition of the microbiome in *N. groenlandicus* and *N. flavomarginatus* across time of day and season in the field. We saw large differences in the most abundant taxa across species and found representatives from genera in both species that are typically endosymbionts, such as *Wolbachia*, *Rickettsia*, and *Rickettsiella* [10, 11, 64]. The difference in bacterial composition between *N. groenlandicus* and *N. flavomarginatus* could be due to the difference in diet. Because *N. groenlandicus* is a sap-sucking insect [38], its microbiome likely contains microorganisms that specifically supply nutrients that are lacking in the sap of the seeds [16]. The diet of *N. groenlandicus* therefore necessitates the presence of specific bacterial symbionts that *N. flavomarginatus* likely does not need because its diet mainly consists of other smaller arthropods [38]. It is therefore not surprising that we found *Candidatus* Schneideria nysicola in *N. groenlandicus* which is an obligate intracellular bacterial symbiont. *Candidatus* S. nysicola is restricted to the seed bug genus where it has been suggested to play an important role in supplying additional essential amino acids to the host that are not present in its sap-based diet [65].

Other symbionts were detected in *N. groenlandicus* in experiment 2, including *Spiroplasma*, *Candidatus* Karelsulcia muelleri, and *Enterococcus*. Like *Candidatus* S. nysicola, *Candidatus* K. muelleri has been found in other sap-feeding insects also belonging to the Hemiptera order, where it has been suggested to play a role in providing nutrients to the host that are otherwise not present in their diet [66]. *Candidatus* K. muelleri has not been reported in *N*. *groenlandicus* before but has been found in the Nearctic leafhopper *Scaphoideus titanus* [67]. Both *Candidatus* S. nysicola and *Candidatus* K. muelleri may therefore positively contribute to the ability of *N. groenlandicus* to live in an environment such as the Arctic with limited resources.

Among the other genera we detected in experiment 1 and 2, some taxa (e.g. *Rickettsiella* and *Rickettsia*) are well documented in insects from polar regions, appearing in several different species of ticks [61, 62] likely acting as endosymbionts [68, 69], while other taxa (e.g. *Spiroplasma* and *Enterococcus*) so far have not been described in Arctic or Antarctic insect species [70]. As for *Wolbachia*, both Holmes et al. [35] and Maistrenko et al. [71] reported that the genus was absent from their Antarctic samples, yet the genus is well presented in the Arctic insects investigated in this study. It thus seems that different polar environments can shape microbial community composition differently which in part may be due to the differences in thermal environment that exists between the two regions [8].

We observed compositional shifts in the bacterial community of both *N. groenlandicus* and *N. flavomarginatus* between sampling days in experiment 1 and between most sampling days across the summer season for *N. groenlandicus* in experiment 2. Similarly, other reports have shown that the gut microbiome of the spring field cricket (*Gryllus veletis*) changed depending on the season, going from autumn to spring [37]. In the current study, environmental variables including temperature, humidity, and food availability may have affected the bacterial community [16, 27, 29]. In accordance with this, we found that the minimum temperature 4 and 8 h prior to sampling, as well as the minimum and maximum humidity 8 h prior to sampling did have a significant effect on the bacterial diversity. Thus, it seems that abiotic factors in the field indeed can influence the bacterial composition of insects, but that this is dependent on the time scale, as we observed no significant differences between morning and evening bacterial composition for each day.

There can be large variation in the bacterial community composition between individuals, which can reduce the ability to establish temporal changes from random changes. However, to reduce the effect of individual variation and our ability to detect differences across time-points, we pooled 5–10 individuals of females for each replicate and carried out two independent experiments to address temporal effects. In addition, all individuals were sampled within the same area of Narsarsuaq town. Adequate sequencing depth was achieved for all experiments (Fig. S1) and we selected the database which returned the highest taxonomic resolution possible. However, we acknowledge that a relatively high proportion of reads remained unassigned, likely reflecting primer-specific biases between sequencing runs (full-length 16S on MinION R9.4.1 in 2021 vs. V1–V8 on PromethION R10.4.1 in 2023). Therefore, we focus our interpretations on within-year comparisons and caution should be taken when making direct comparisons between sampling years.

### Influence of Biotic and Abiotic Factors on Bacterial Community Composition in Laboratory Settings

Several studies have suggested that the microbiome may play an important role for species thermal tolerance (see e.g. Lemoine et al. [26]), but, to our knowledge, few studies have addressed this in the field. We found that the temperature prior to sampling in the field affected the microbiome, and through controlled laboratory experiments we showed how acclimation temperature affects the bacterial composition in *N. groenlandicus* and *N. flavomarginatus*. For both species, we found that *Rickettsia*, *Spiroplasma* and *Wolbachia* were present in the hosts’ microbiome. These bacterial symbionts have previously been linked to thermal tolerance in invertebrates, such as in the spider mite (*Tetranychus truncatus*) [72] and chestnut weevil (*Curculio sikkimensis*) [73]. It is thus possible that the bacterial symbionts offer their host some adaptative advantage to the ambient temperature [73], which could prove helpful in environments as thermally variable as the Arctic.

When investigating the influence of humidity on the bacterial community in *N. flavomarginatus*, we saw that while there were no significant changes in microbial diversity, all humidities grouped independently signifying an effect on the microbial composition. This further supports our findings from experiment 2, where we observed that both the lowest and highest recorded humidities prior to sampling affected the microbial composition. In this experiment, the bacterial symbionts *Wolbachia*, *Rickettsia*, *Rahnella*, and *Spiroplasma* were all detected in *N. flavomarginatus* (Fig. S6). Of these, only *Wolbachia* has been linked to dehydration tolerance in invertebrates [74]. *Clavibacter michiganensis* and *Candidatus* S. nysicola were also detected in *N. flavomarginatus*. As *C. michiganensis* is a plant pathogen [75] and *Candidatus* S. nysicola is proposed to be involved in nutrient provisioning in *Nysius* species [65], which *N. flavomarginatus* feed on, it is possible that these two bacteria were simply transferred to *N. flavomarginatus* through its diet.

Several studies have shown how diet can influence the gut microbiome and that changes in the host microbiome can happen within less than 24 h [17, 20, 76], but few have attempted to link variation under natural conditions to specific changes associated with changed diets under laboratory conditions. We found that individuals of *N. flavomarginatus* that were fed with *N. groenlandicus* grouped close to individuals of *N. groenlandicus* compared to other diets, indicating that bacteria may have been transferred from prey (*N. groenlandicus*) to predator (*N. flavomarginatus*) (see also Fig. S8). This corresponds with findings by Saqib et al. [20] who found that the gut microbiome of spiders was influenced by their prey and the diet of their prey. For both *N. flavomarginatus* and *N. groenlandicus* we found that the bacterial community composition was different between all diets. This corresponds with findings by Luo et al. [17] which saw that different diets changed the composition as well as the abundance of the gut microbiome in a species of mirid bug (*Adelphocoris suturalis*). This is probably because different diets require different types of microorganisms to aid in breakdown of the food and, if needed, supplementation of missing nutrients in the diet.

## Supplementary Information

Below is the link to the electronic supplementary material.

Supplementary file1 (PDF 2548 KB)

## Data Availability

The data supporting this article is available at Zenodo (10.5281/zenodo.18171991).
