# Supplementary material for: Arctic Insects Show a Highly Dynamic Microbiome Shaped by Abiotic and Biotic Variables
Source: Microb Ecol. 2026 Jan 10;89(1):43. doi: 10.1007/s00248-025-02685-z (PMC12860762; doi:10.1007/s00248-025-02685-z)
Supplement: Supplementary file 1 — Supplementary file1 (PDF 2548 KB) [file 248_2025_2685_MOESM1_ESM.pdf]

Title: Arctic insects show a highly dynamic microbiome shaped by abiotic and biotic variables

Journal: Microbial Ecology

Authors: Sara Nørris Christoffersen[a*], Stine Karstenskov Østergaard[a], Nadieh de Jonge[a], Cino Pertoldi[a,b], Jesper Givskov Sørensen[c], Natasja Krogh Noer[d], Torsten Nygård Kristensen[a], Jeppe Lund Nielsen[a], and Simon Bahrndorff[a]

[a]*Department of Chemistry and Bioscience, Aalborg University, Aalborg, Denmark*

[b]*Aalborg Zoo, Aalborg, Denmark*

[c]*Department of Biology, Aarhus University, Aarhus, Denmark*

[d]*ENORM Biofactory, Flemming, Denmark*

*__Corresponding author: Sara Nørris Christoffersen__, Aalborg University, Department of Chemistry and Bioscience, Fredrik Bajers Vej 7H, 9220 Aalborg East, e-mail: saranc@bio.aau.dk

Supplementary Materials

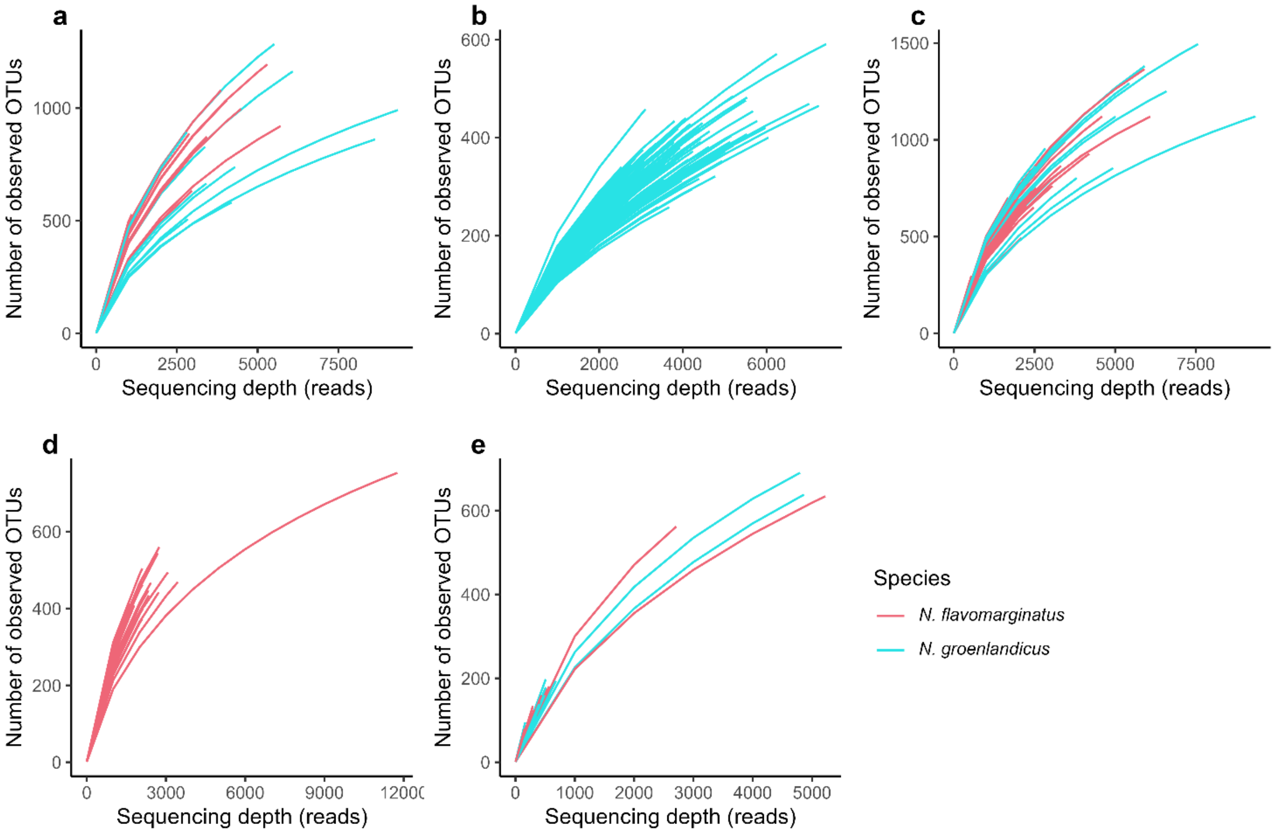

Fig. S1 Rarefaction curve for experiment 1 (a), 2 (b), 3 (c), 4 (d), and 5 (e) with number of reads per sample (x-axis) and number of unique OTUs per sample (y-axis).

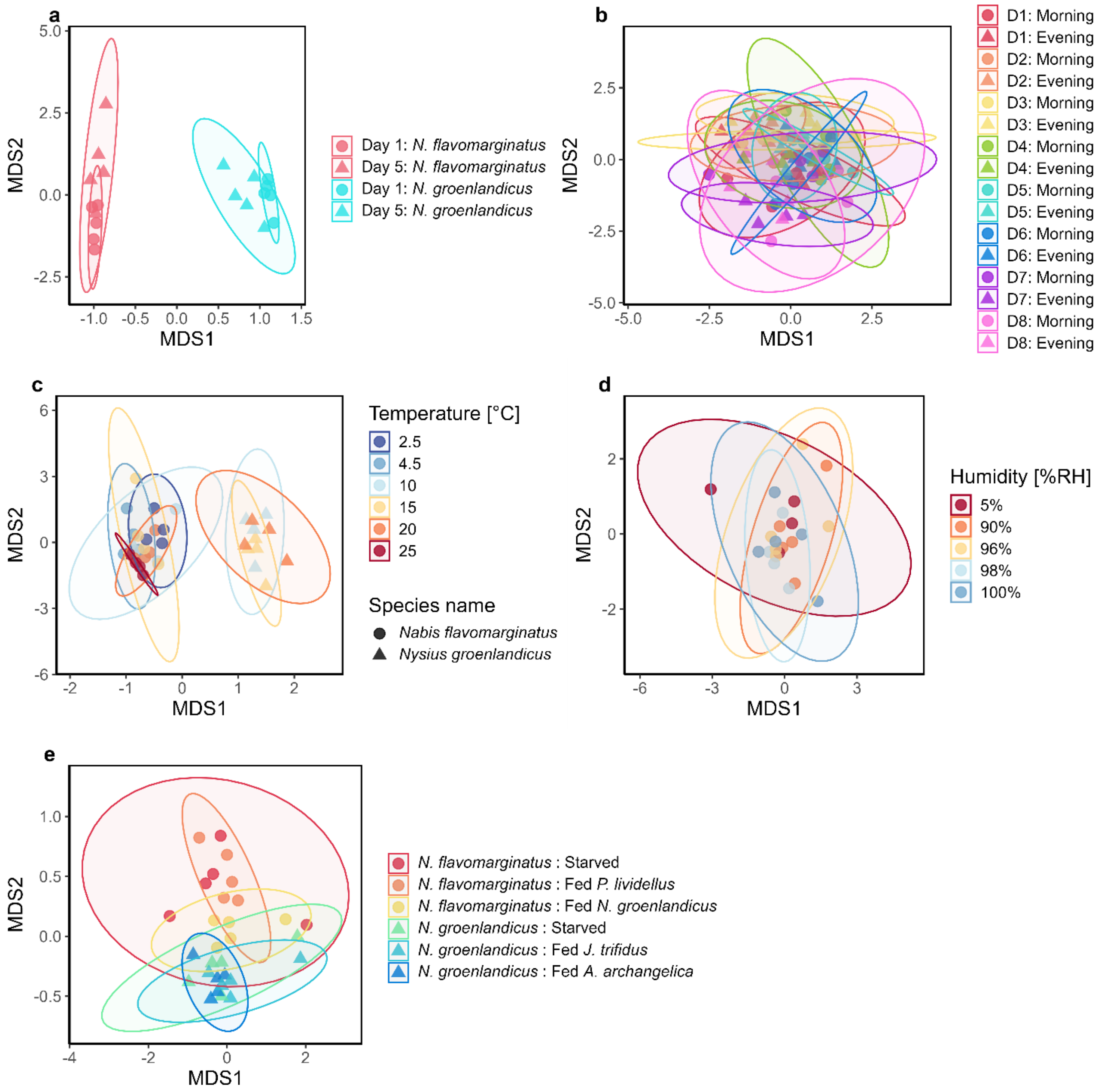

Fig. S2 NMDS with Bray-Curtis distances for experiment 1 (a), 2 (b), 3 (c), 4 (d), and 5 (e).

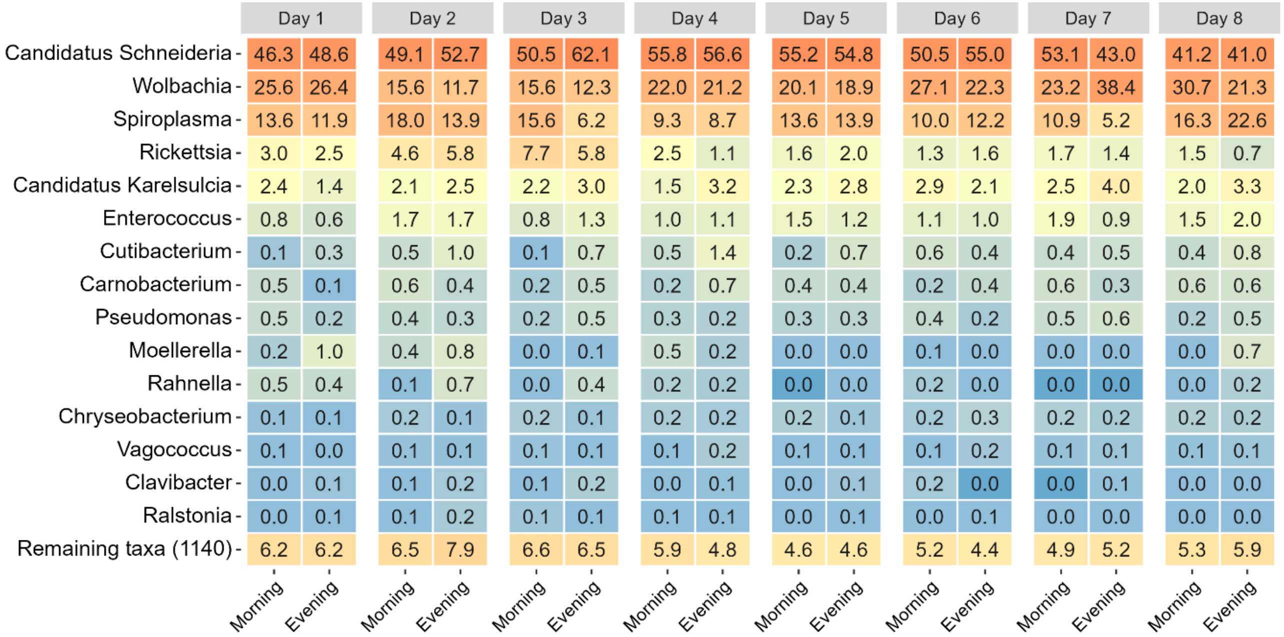

Fig. S3 Heatmap of the relative abundance of the top 15 genera in *Nysius groenlandicus* in experiment 2, faceted by sampling day and time.

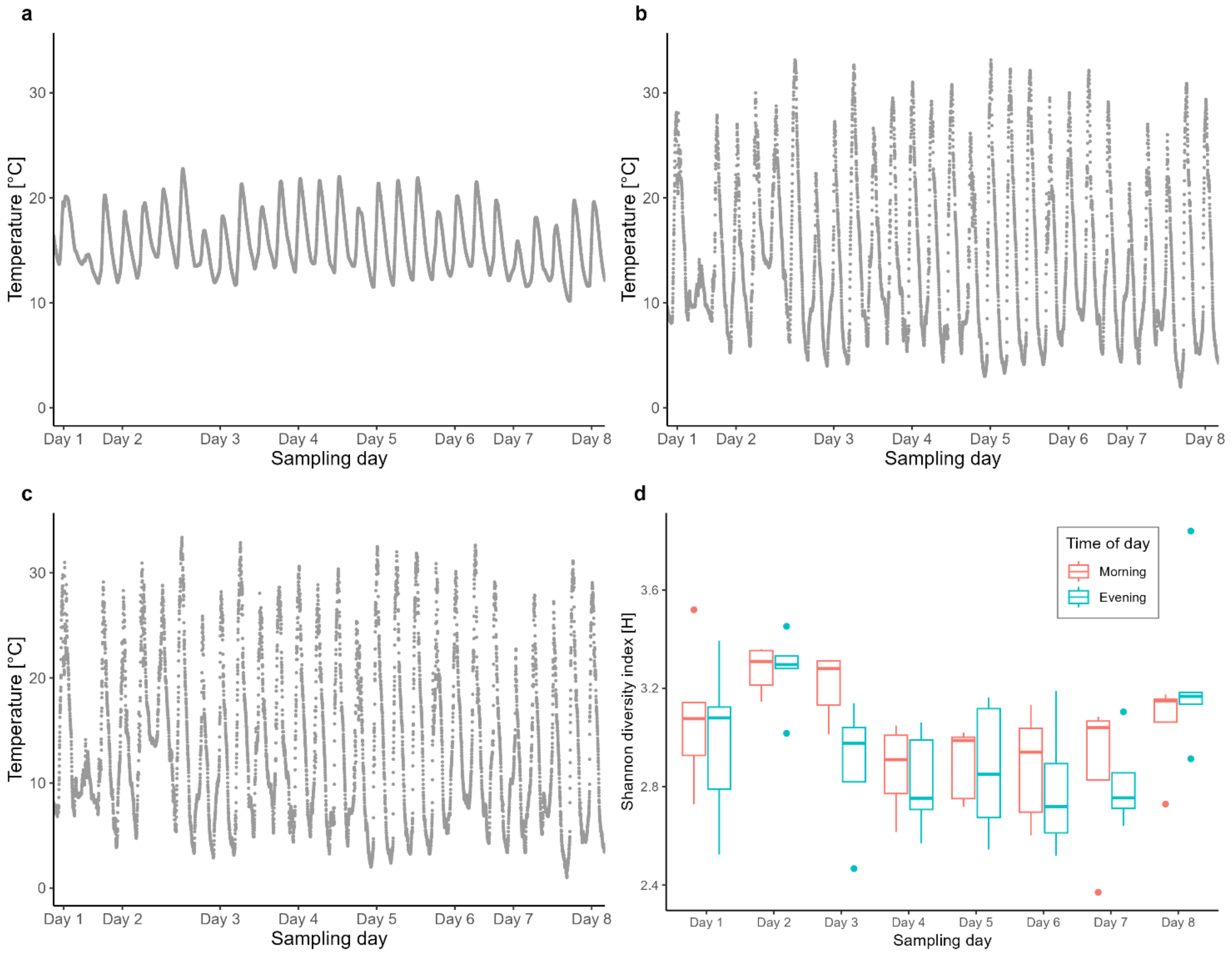

Fig. S4 Temperature in the field measured in the soil (a), on the surface of the soil (b), and in the air (c) across the sampling period in experiment 2. (d) Bacterial diversity as measured by the Shannon diversity index across the sampling period in experiment 2.

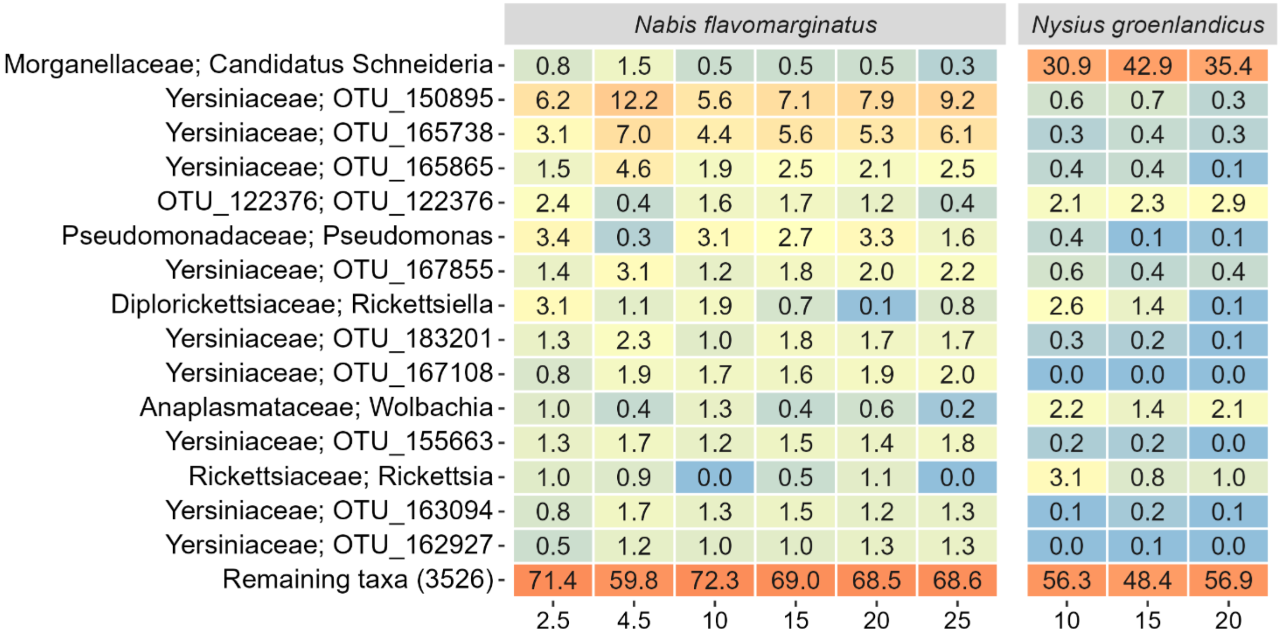

Fig. S5 Heatmap of the relative abundance of the top 15 shared OTUs (at family and genus level) between *Nabis flavomarginatus* and *Nysius groenlandicus* in experiment 3, faceted by acclimation temperature.

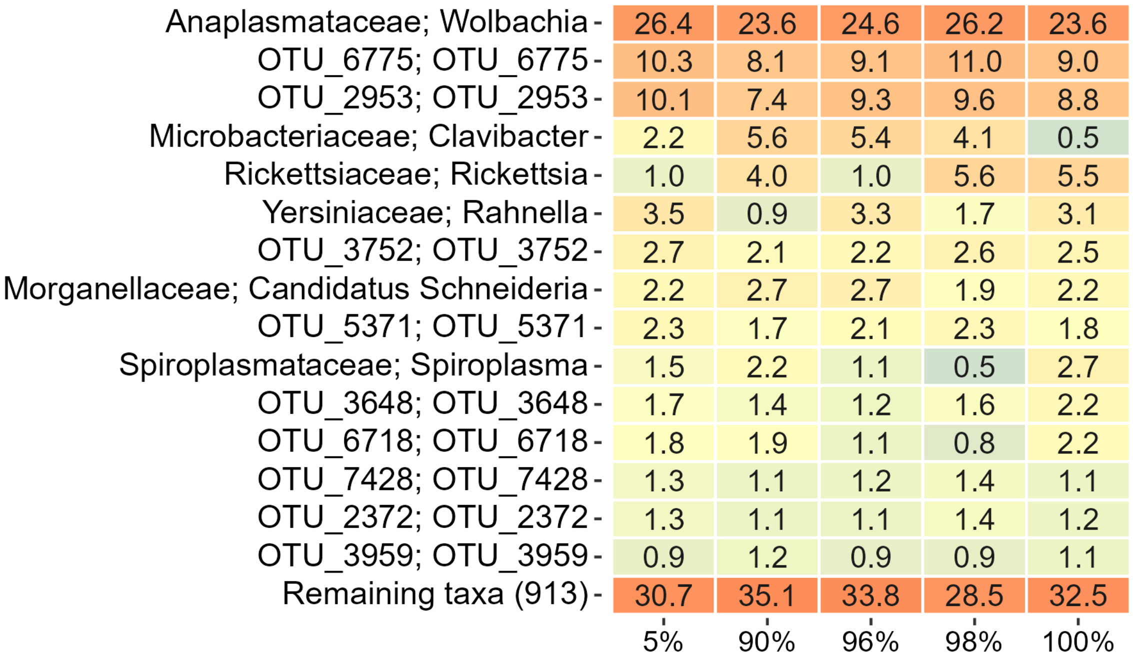

Fig. S6 Heatmap of the relative abundance of the top 15 OTUs (at family and genus level) in *Nabis flavomarginatus* in experiment 4, faceted by humidity.

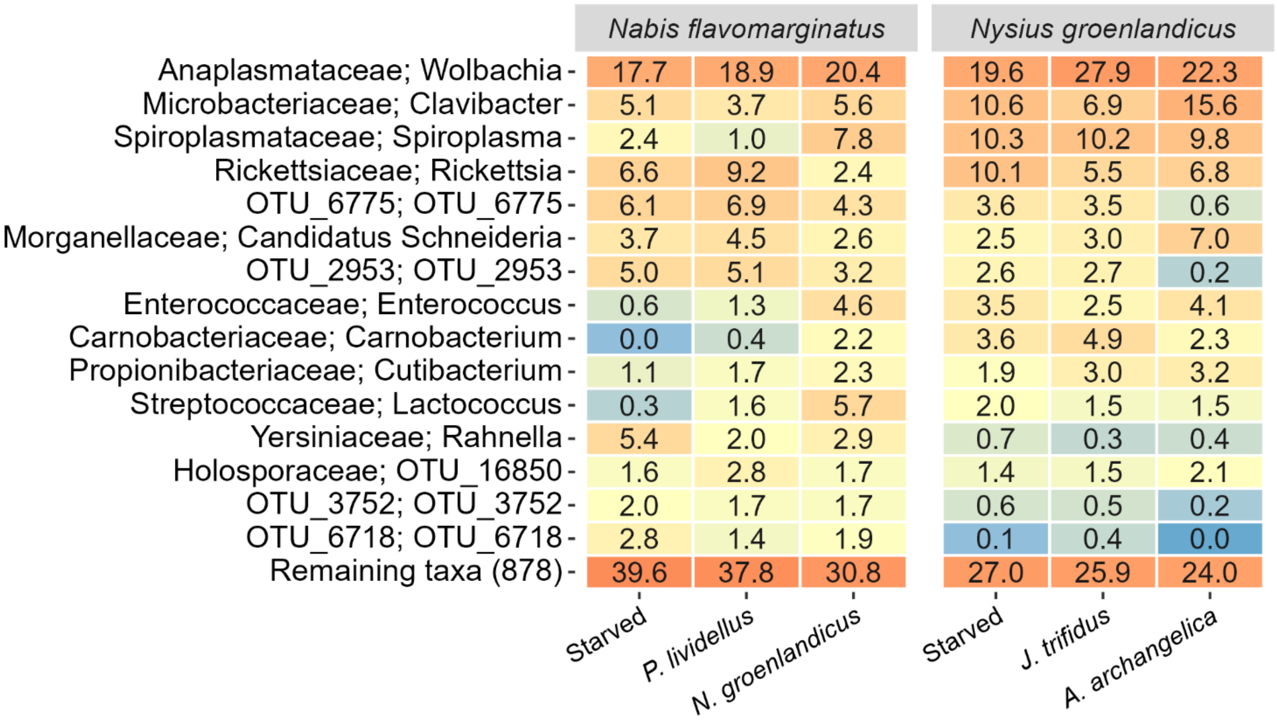

Fig. S7 Heatmap of the relative abundance of the top 15 shared OTUs (at family and genus level) between *Nabis flavomarginatus* and *Nysius groenlandicus* in experiment 5, faceted by diet.

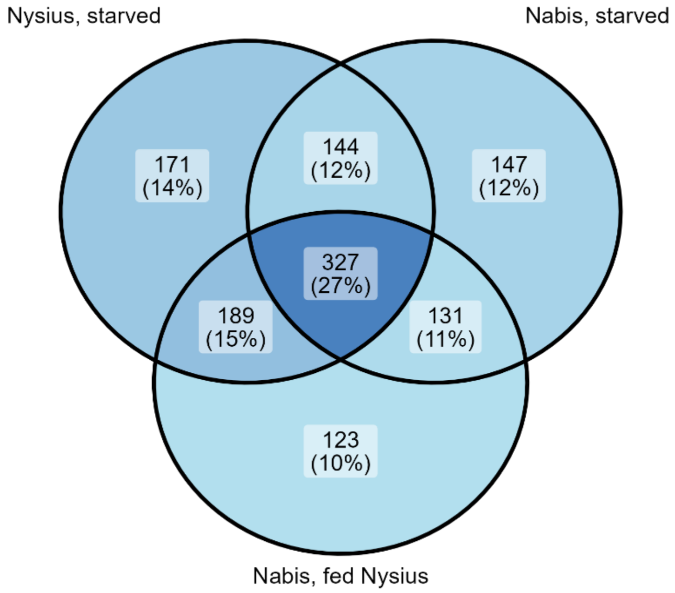

Fig. S8 Venn diagram showing the count (and percentage) of shared OTUs between three groups from experiment 5; *Nysius groenlandicus* that have been starved (top left), *Nabis flavomarginatus* that have been starved (top right), and *N. flavomarginatus* that have been fed with *N. groenlandicus* (bottom).

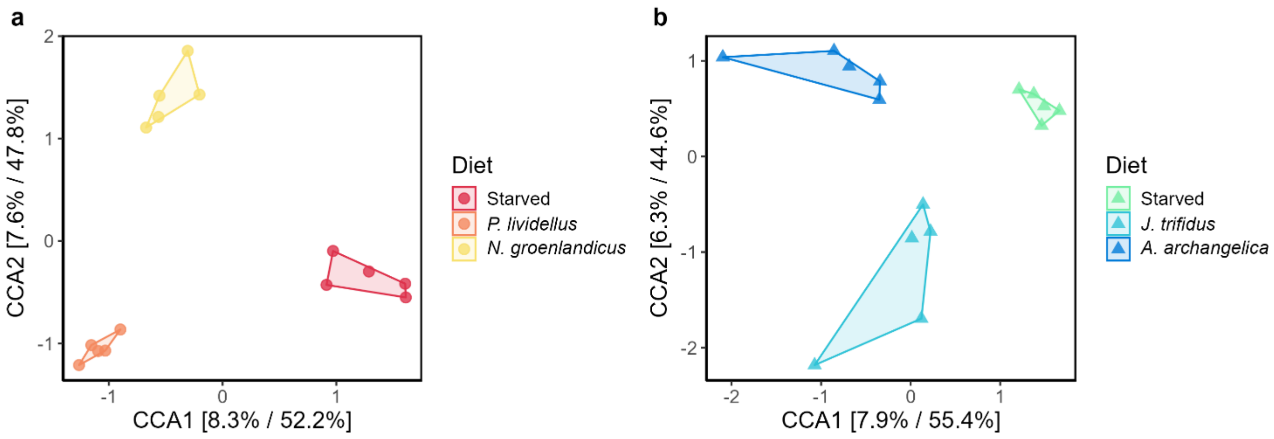

Fig. S9 Canonical Correspondence Analysis (CCA) for *N. flavomarginatus* (a) and *N. groenlandicus* (b) in experiment 5.
